# A Game Model for Medical Service Pricing Based on the Diagnosis Related Groups

**DOI:** 10.3389/fpubh.2021.737788

**Published:** 2021-11-30

**Authors:** Jinli Duan, Zhibin Lin, Feng Jiao

**Affiliations:** ^1^College of Modern Management, Yango University, Fuzhou, China; ^2^Durham University Business School, Durham University, Durham, United Kingdom; ^3^INTO Newcastle University, Newcastle University, Newcastle upon Tyne, United Kingdom

**Keywords:** bargaining model, pricing, government compensation, medical service, patient, quality

## Abstract

**Background:** Currently there are various issues that exist in the medical institutions in China as a result of the price-setting in DRGs, which include the fact that medical institutions tend to choose patients and that the payment standard for complex cases cannot reasonably compensate the cost.

**Objective:** The main objective is to prevent adverse selection problems in the operations of a diagnosis-related groups (DRGs) system with the game pricing model for scientific and reasonable pricing.

**Methods:** The study proposes an improved bargaining game model over three stages, with the government and patients forming an alliance. The first stage assumes the alliance is the price maker in the Stackelberg game to maximize social welfare. Medical institutions are a price taker and decide the level of quality of medical service to maximize their revenue. A Stackelberg equilibrium solution is obtained. The second stage assumes medical institutions dominate the Stackelberg game and set an optimal service quality for maximizing their revenues. The alliance as the price taker decides the price to maximize the social welfare. Another Stackelberg equilibrium solution is achieved. The final stage establishes a Rubinstein bargaining game model to combine the Stackelberg equilibrium solutions in the first and second stage. A new equilibrium between the alliance and medical institutions is established.

**Results:** The results show that if the price elasticity of demand increases, the ratio of cost compensation on medical institutions will increase, and the equilibrium price will increase. The equilibrium price is associated with the coefficient of patients' quality preference. The absolute risk aversion coefficient of patients affects government compensation and total social welfare.

**Conclusion:** In a DRGs system, considering the demand elasticity and the quality preference of patients, medical service pricing can prevent an adverse selection problem. In the future, we plan to generalize these models to DRGs pricing systems with the effects of competition of medical institutions. In addition, we suggest considering the differential compensation for general hospitals and community hospitals in a DRGs system, in order to promote the goal of hierarchical diagnosis and treatment.

## Introduction

The National Health Commission and the other relevant departments in China have started to test the diagnosis-related groups (DRGs) system in 30 pilot locations since 2019. DRGs refer to a price standardization mechanism based on patients' classification. Patients can be classified based on their demographic and medical information, such as age, gender, length in hospital, types of diseases, the severity of diseases and comorbidity, complications, the necessity of surgery, etc (1). DRGs aim to categorize patients who have similar clinical features and medical resource consumption into the same groups, which can standardize prospective payment to hospitals or other medical institutions (2).

Currently there are various issues that exist in medical institutions around the world as a result of the price-setting in DRGs, which include the fact that medical institutions tend to choose patients, the payment standard for complex cases cannot reasonably compensate the cost, and medical service providers increase the amount of outpatient services in order to reduce hospitalization expenses (3). High price-setting in DRGs leads to the difficulty of the government controlling medical expenses, while low price-setting is not able to fairly compensate the costs in medical institutions (1), and even leads to hospitals refusing to accept patients in critical conditions. This conflict in price-setting can be understood as a problem of adverse selection (4). Therefore, it is imperative to explore an optimal pricing system that helps to resolve the problem of adverse selection.

Many scholars apply the game theory to the pricing of medical services, which solves the conflict of interests of the stakeholders in the pricing of medical services (5–10). For example, Weingarten (11) applies a generalized game theory model to study the price-setting strategy of competitive medical institutions. Westhoff et al. (5) and Agee et al. (8) apply game theory to study cooperation and competition in disease treatment payment and compensation. Nagurney (12) focus on the supply chain of blood products in an asymmetric information environment. Their study determines a generalized Nash equilibrium model by adopting sharing constraints. This model discusses the price-setting problem in the blood products supply chain, which finds that participants nearer the termination of the supply chain will have better access to information and stronger bargaining power. However, most of these studies focus on the linkages between price makers and medical institutions' behavior, which ignore the impact on medical buyers' behavior and patient preferences with regard to medical service quality.

The present study thus aims to address the above issues by providing some improvements in the game theoretical model assumptions and the model design. First, the quality of medical service is introduced as a variable in a medical service pricing game model. Second, in China, medical institutions are monopolistic in the provision of medical services, and they are in a dominant position in the negotiation of price and quality. Patients as individuals, their bargaining power is scattered. Moreover, the lack of professional knowledge and complete information also weaken patients' bargaining power. Therefore, it is necessary for the government, as the co-payer of medical services, to balance the power of the supplier and the purchasers.

We design a DRG pricing and compensation model as a composite model of two Stackelberg games and bargaining games. In the first round of the game, with the alliance of government and patients as the leader of the game and the medical institutions as the game follower, we get an equilibrium solution about the price and quality of medical services. In the second round of the game, with the medical institutions as the leader of the game and the alliance of government and patients as the follower of the game, we get another equilibrium solution about the price and quality of medical services. In the third round of the game, the two equilibrium solutions obtained above are used as the initial solution of the bargaining game, and the government and patients share the medical expenses.

## Related Works

In the early days, scholars generally considered the competitiveness of the medical service market and established a game pricing model. The Shleifer pricing mechanism is a type of yardstick regulation developed by Shleifer (13). They show that the specific Nash equilibrium is the point where medical institutions achieve optimal marginal cost. Ellis and McGuire (14) explore an optimal neutral risk situation for medical insurance paying to medical institutions. Allen and Gertler (15) use game theory to study the influence of price-setting strategy on patients' satisfaction with services. Weingarten (11) applies a generalized game theory model to study the price-setting strategy of competitive medical institutions. Westhoff et al. (5) and Agee et al. (8) apply game theory to study cooperation and competition in disease treatment payment and compensation. Robinson (16) adopt a Stackelberg game theory model in discussing the relationship between cost transfer and payment methods for medical institutions. They reveal a direct connection between the cost-shifting and medical service payment policy, which shows paying by classification of diseases is better than paying by medical service items.

Many other scholars develop game models on price-setting and negotiation by multiple participants in the medical services. Most of these studies are based on information asymmetry between participants (17, 18). Yaesoubi and Roberts (19) develop a game model that considers two types of constraints. The needed constraint is the bargaining powers of medical service purchasers, and the soft constraint is the cost reimbursement for service providers. These two types of constraints help the model better determine the pricing in an information asymmetric environment. De (20) adopts game theory to examine the impact of information transactions on the relationship between medical institutions and patients, and finds that there will be a Nash equilibrium when patients can restrict medical suppliers' induced demand.

In recent years, scholars have focused on the influence of various factors, such as government intervention (21, 22), medical service quality (17, 23–25), and payment mode of medical services (26, 27), on the results of pricing games (2, 28–30). Behzad and Jacobson (31) investigate the impact of government price intervention and duopoly competition on medical price-setting. Their study applies the asymmetric Bertrand-Edgeworth model to study this impact within a duopoly market. A similar study developed by das Chagas Moura et al. (32), who apply queue theory to improve the Stackelberg game model, focuses on the problems of medical service pricing and medical quality. Koenecke (33) analyses the non-extreme equilibrium between two different payment methods in medical insurance and medical institutions by applying a Stackelberg game model. These two payment methods include a charge on a per-service basis and a per-patient basis. Assuming medical insurance can offer non-linear bonuses, the analysis shows that charges on a per-patient basis can better contribute to sharing medical service information between medical institutions and patients.

## The Novel Bargaining Game Model

### Model Assumptions

Assuming there are three players in the medical service market, including the government, medical service suppliers, and patients. Patients have only one treatment demand on each type of disease and pay for a certain ratio of treatment fee to acquire effective medical services. Medical institutions can solve patients' queuing problems by having an appointment booking system. As payers of medical service, the government and patients form an alliance.

Assuming medical institutions have sufficient ability to fulfill patients' demands, the decision variable of the alliance is the treatment price of each disease, and the objective function is total social welfare. The decision variable of medical institutions is medical service quality, and the objective function is the profit of medical service. If assuming the demand function of a medical service is a linear function of the medical service price and quality, which is *Q* = *a* − *bp* + *cq*, where *a, b, c* ≥ 0, *b* is the price elasticity of demand (PED) for a single type of disease, *c* is the coefficient of patients' preference on quality, and *p* and *q* are decision variables. Moreover, the cost function of a medical service is a function of the medical service quality, which is $C=F+\frac{1}{2}\gamma q^{2}Q$, where *F* is the fixed cost of medical service, $\frac{1}{2}\gamma q^{2}Q$ is the marginal cost, and γ is the coefficient of quality cost.

The objective function of the alliance is the social total surplus, which refers to consumer surplus plus producer surplus.


(1) $$\begin{array}{l} WF=\int_{0}^{Q}\frac{a+cq-Q}{b}dQ-pQ+pQ-F-\frac{1}{2}\gamma q^{2}Q\\ \;=\int_{0}^{Q}\frac{a+cq-Q}{b}dQ-F-\frac{1}{2}\gamma q^{2}Q \end{array}$$

And the utility of patients is


(2) $$\begin{array}{l} Up=\int_{0}^{Q}\frac{a+cq-Q}{b}dQ-pQ \end{array}$$

The profit of medical institutions is


(3) $$\begin{array}{l} \pi =pQ-F-\frac{1}{2}\gamma q^{2}Q \end{array}$$

### Model Formation

The bargaining game on disease treatment pricing will be developed between the alliance and the medical institutions. In the first stage, the alliance will be a price maker and determine a pricing strategy for treatment based on social welfare maximization. Medical institutions will the price taker to determine a service quality strategy for responding to the price strategy from the alliance. A Stackelberg equilibrium solution will be achieved to balance the treatment price and service quality. In the second stage, medical institutions will be the price maker and determine an optimal service quality strategy for maximizing their profits. While the alliance as the price taker will determine another optimal treatment price strategy based on social welfare maximization in response to the medical institutions' strategy. Another Stackelberg equilibrium solution will be achieved in this stage to balance treatment price and service quality. These two equilibrium solutions will be an initial solution for the bargaining model between the alliance and medical institutions in the final stage. The model will be in the Stackelberg game based on the bargaining power of each player and it will achieve a final Stackelberg bargaining equilibrium solution.

#### The First Round Stackelberg Game

The first stage is to apply the Stackelberg model to analyze the pricing behavior by the alliance as a price maker. Based on social total surplus maximization, the alliance can determine a price *p*_1_ for a certain medical treatment, and medical institutions as price takers will determine a maximum profit on this certain treatment which is *q*_1_ based on *p*_1_.

By applying the reverse acquisition, firstly, when maximizing the medical institutions' profit,


(4) $$\begin{array}{l} \frac{\partial \pi }{\partial q_{1}}=\frac{\partial \left(p_{1}Q-F-\frac{1}{2}\gamma {q_{1}}^{2}Q\right)}{\partial q_{1}}=0 \end{array}$$

And we obtain:


(5) $$\begin{array}{l} q_{1}=\frac{rb_{1}p_{1}-ar+\sqrt{{\left(rb_{1}p_{1}-ar\right)}^{2}+6c^{2}rp_{1}}}{3cr} \end{array}$$

Plugging (5) into (1), where the utility function maximization for the alliance will be:


(6) $$\begin{array}{l} \frac{\partial WF}{\partial p_{1}}=\frac{\partial \left(\int_{0}^{Q}\frac{a+cq_{1}-Q}{b}dQ-F-\frac{1}{2}\gamma {q_{1}}^{2}Q\right)}{\partial p_{1}}=0 \end{array}$$

We then have the following:


(7) $$\begin{array}{l} p_{1}=\frac{\gamma^{2}b^{2}}{9c^{2}}+\frac{\gamma b-2a-3}{3b} \end{array}$$

Plugging (7) into (5) and we get:


(8) $$\begin{array}{l} q_{1}=\frac{b\gamma \sqrt{{\left(a\gamma -b\right)}^{2}-6c^{2}}}{3ac} \end{array}$$

**Lemma 1**. *if and only if* $F\geq \frac{b\left(a+\gamma^{2}\right)}{ac-\gamma }$, *there is an equilibrium solution that exists for the pricing and quality Stackelberg game model between the alliance and medical institutions, and this equilibrium solution is* $p_{1}=\frac{\gamma^{2}b^{2}}{9c^{2}}+\frac{\gamma b-2a-3}{3b}$, $q_{1}=\frac{b\gamma \sqrt{{\left(a\gamma -b\right)}^{2}-6c^{2}}}{3ac}$

The ratio between the fixed cost and marginal cost in medical institutions will be the minimum level of government compensation, and it will be a positive relationship with PED. In other words, when the PED of a certain disease treatment increases, the ratio of cost compensation on medical institutions will increase, and the treatment price will increase. Whereas, when the ratio of cost compensation on medical institutions decreases, the treatment price will decrease. This is the opposite of Ramsey pricing because of the welfare in medical services. When a type of disease belongs to the basic medical service, PED decreases and the ratio of compensation on medical services will decrease. While if a type of disease is not listed as a basic medical service, such as rehabilitation, when PED increases, the ratio of compensation on medical services will increase.

#### The Second Round Stackelberg Game

In this round of Stackelberg games, medical institutions are the first decision-makers, and the government and patients' alliance are the followers. Based on the objective of profit maximization, medical institutions will determine the decision variable *q*_2_ for optimizing the service quality. The alliance then determines an optimal price *p*_2_ for responding to the decision by the medical institutions. By applying the reverse acquisition, a price for the alliance can be obtained, which guarantees a maximized utility. Based on ∂WF∂p2=0, it can achieve


(9) $$\begin{array}{l} p_{2}=\frac{1}{2}c{q_{2}}^{2}+\frac{cq_{2}+a-1}{b} \end{array}$$

Plugging (9) into (3), based on $\frac{\partial \pi }{\partial q_{2}}=0$, it can achieve


(10) $$\begin{array}{l} q_{2}=\frac{bc\sqrt{a\gamma -b+6\gamma^{2}}}{3a} \end{array}$$

Plugging (10) into (9), it can achieve


(11) $$\begin{array}{l} p_{2}=\frac{1}{2}b^{2}c^{3}\frac{a\gamma -b+6\gamma^{2}}{9a^{2}}+c^{2}\sqrt{a\gamma -b+6\gamma^{2}}+\frac{a-1}{b} \end{array}$$

**Lemma 2**. *if and only if* $\sqrt{\frac{\gamma^{2}-a\gamma }{4a}}<c<\sqrt{\frac{\gamma^{2}+a\gamma }{4a}}$, *there is an equilibrium solution that exists in the Stackelberg game on the pricing and quality for the alliance and medical institutions. This solution is as follows*:


$$\begin{array}{l} p_{2}=\frac{1}{2}b^{2}c^{3}\frac{a\gamma -b+6\gamma^{2}}{9a^{2}}+c^{2}\sqrt{a\gamma -b+6\gamma^{2}}+\frac{a-1}{b},\\ \;q_{2}=\frac{bc\sqrt{a\gamma -b+6\gamma^{2}}}{3a} \end{array}$$

If the coefficient of patients' quality preference is in a certain range, the equilibrium price of disease treatment will increase with the increasing of the coefficient of patients' quality preference. Because the larger the coefficient of patients' quality preference, the more competitive medical service quality is, and that is, if a medical institution slightly improves the quality of medical services, it will lead to a large increase in the demand for medical services. This will also lead to an increase in the market share, which brings more profits to medical institutions. In this regard, medical institutions normally set their treatment price high. However, if the coefficient of patients' quality preference goes beyond a certain range, especially at a very high level, the treatment price will no longer be the priority in patients' consideration. To achieve effective treatment without waiting in a queue will be the first thing considered by the patients. In this situation, the treatment price will lose the ability to balance market supply and demand. Opposite to this, if the coefficient of patients' quality preference is low, the service quality competition among medical institutions will be ineffective. Patients are not sensitive to the quality of medical services. If they feel that the quality of medical services is the same, they will be sensitive to the price. The market share of which medical institution is cheap will be large. Therefore, medical institutions will adopt a low-price strategy.

The alliance as the price maker in the Stackelberg game determines a medical pricing strategy, medical institutions as the price taker have to respond to the strategy and make an optimal service quality strategy. The alliance has the first-mover advantage. When medical institutions are the price maker, the alliance as the price taker has to respond by determining an optimal medical pricing strategy. In this regard, medical institutions have the first-mover advantage.

To satisfy the needs of the initial solution for the bargaining model, the price level of unit quality for the equilibrium solutions (*p*_1_, *q*_1_) and (*p*_2_, *q*_2_) are standardized as p1*=p1q1 and p2*=p2q2.

#### The Bargaining Game for Co-payment Medical Expenses

A bargaining range $\left(p_{1}^{*},p_{2}^{*}\right)$ made by the medical institutions and the alliance is in a game. By applying the Rubinstein bargaining model, an equilibrium solution is achieved


$$\begin{array}{l} \bar{p}=p_{1}^{*}+\frac{1-\theta 2}{1-\theta 1\theta 2}p_{2}^{*} \end{array}$$

Where θ1, θ2 are the discount factors for the alliance and medical institutions, respectively. θ1 often depends on the patience level of the alliance in negotiation, the opportunity cost of medical treatment, and the severity of the diseases. θ2 depends on the patience level of medical institutions in negotiation, difficulty of patient referral, and the difficulty and risk of treatment.

Through the bargaining game between the alliance and medical institutions, the medical service is priced for the alliance as $\bar{p}$. In this second game, the government and patients will get in the game of sharing medical payments based on the price $\bar{p}$. The government and patients share the payment for the medical service through a third-party payment mechanism, i.e., health insurance. Paying through health insurance can easily put patients into moral hazard, because patients may be insensitive to medical prices and overspend on treatment. The early studies by Arrow (34) and Zeckhauser (35) find that health insurance should not fully cover all the medical cost. The effective payment method is to set a reasonable co-payment ratio, which diversifies the risks of patients and balances risks of patients' moral hazard and the profits of the insurance business. Ellis and Manning (36) and Eeckhoudt (37) state that the marginal profit achieved from the risk diversification by insurance equals their marginal cost in moral hazard; the health insurance co-payment ratio is the optima. Blomqvist (38) discusses the optimal design of health insurance co-payment ratio in a non-linear model and finds that the optimal health insurance co-payment ratio depends on patients' health level.

The medical pricing bargaining model was developed in the first stage by the alliance and medical institutions, which aims to prevent moral hazard caused by patient's excessive consumption of medical care. The medical treatment co-payment ratio game model developed in the second stage aims to prevent the overtreatment risk caused by the third-party payment system.

Based on the result in the first stage, by adding the co-payment ratio λ, the demand of medical function will be *D* = *a* − *bλp*, which refers to a linear function of patients' medical demand, where *a* ≥ 0, *b* ≥ 0 and 0 ≤ λ ≤ 1.

Patients' moral hazard in health insurance can cause waste in social welfare. Therefore, this study considers that the optimal co-payment rate is if and only if the social welfare loss caused by medical insurance is minimized. Three parts of health insurance may cause a loss in social welfare. The first part is the deadweight loss caused by patients' moral hazard. Based on Arrow's study, the deadweight loss caused by patients' moral hazard is (1−λ)2b2. The second part is the government's expectation of opportunity cost on its medical support. This can be presented as $\delta \left[\left(1-\lambda \right)\left(a-b\lambda \right)\right]\overset{-}{p}$. The third part is the cost of risk prevention, which is presented as Rλ2σ22, where δ is the incidence of diseases, *R* is the coefficient of patients' absolute risk aversion, and σ^2^ is the variance of the medical treatment price.

Therefore, the government's utility function is the total loss of social welfare.


(12) $$\begin{array}{l} \min WL=\frac{1}{2}{\left(1-\lambda \right)}^{2}b+\delta \left[\left(1-\lambda \right)\left(a-b\lambda \right)\right]\bar{p}+\frac{1}{2}R\lambda^{2}\sigma^{2} \end{array}$$

By deriving (12), it will achieve


(13) $$\begin{array}{l} \frac{\partial WL}{\partial \lambda }=-\left(1-\lambda \right)b-b\delta \overset{-}{p}\left(1-\lambda \right)-\delta \overset{-}{p}\left(a-b\lambda \right)+R\lambda \sigma^{2}=0 \end{array}$$

Solving (13) will achieve


(14) $$\begin{array}{l} \lambda^{*}=\frac{b+\delta \overset{-}{p}\left(a+b\right)}{b+2b\delta \overset{-}{p}+R\sigma^{2}} \end{array}$$

**Lemma 3**. *if and only if* $R\geq \frac{\sigma^{2}b+\frac{1}{2}a\delta }{\delta^{2}a}$, *the loss of total social welfare decreases, then increases with the increase of social health insurance co-payment ratio. If* $R<\frac{\sigma^{2}b+\frac{1}{2}a\delta }{\delta^{2}a}$, *the loss of total social welfare decreases with the increase of social health insurance co-payment ratio*.

The coefficient of patients' absolute risk aversion stands for the ability of patients to predict the incidence and loss caused by diseases. If the coefficient is lower than a certain threshold value, an increase in the social medical insurance co-payment ratio will bring about a reduction in the total social welfare loss, which is an increase in the total social welfare. In contrast, if the coefficient is higher than the certain threshold value, the increase of social health insurance co-payment ratio will reduce the loss of total social welfare first and then increase, which means that the total social welfare first increases and then decreases.

## Results and Discussion

The above section presents the bargaining game model on pricing and government compensation between the government, patients, and medical institutions. In the first stage, the alliance formed by the government and patients leads the price as the price maker. The alliance will determine a pricing strategy bases on social welfare maximization. Medical institutions as price takers will make a service quality strategy in response to the pricing strategy from the alliance. This will form a Stackelberg equilibrium solution between prices of disease treatment and medical service quality.

In the second stage, medical institutions will be the price maker in the game. Based on profit maximization, medical institutions will create an optimal service strategy for providing the highest level of medical quality. While based on the social welfare maximization, the alliance will be the price taker, to make an optimal disease treatment price in response to the optimal service strategy made by the medical institutions. This will create another Stackelberg equilibrium solution between prices of disease treatment and medical service quality.

**Figure 1:**
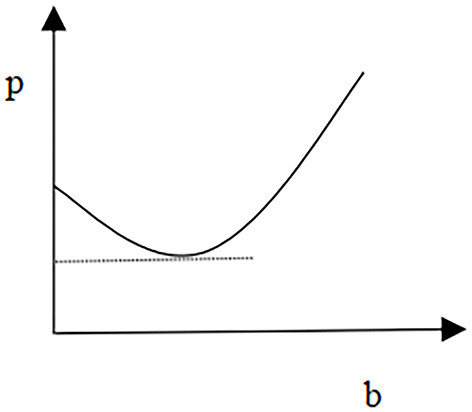
Price and PED.

Taking these two equilibrium solutions as the initial solution for the bargaining game between the alliance and medical institutions, and based on the bargaining power of the alliance and medical institutions, we can achieve an equilibrium price of Rubenstein's bargaining game through developing Rubenstein's game. In the final stage, based on the minimization of deadweight social loss, the government and patients will share the payment of medical costs. To analyze PED on diseases, the coefficient of patients' preferences on service quality, the coefficient of patients' absolute risk aversion, and the relationship between disease pricing and government compensation, this study provides several specific values to simulate a series of impacts on the equilibrium price. Firstly, the study analyzes the PED on the equilibrium price. It assumes that the coefficient of the demand function *a* = 0.3, the coefficient of disease treatment quality cost γ = 10, and the coefficient of patients' preferences on treatment quality *c* = 0.6. Figure 1 shows the relationship between the equilibrium price and disease PED.

The relationship presented in Figure 1 is in a scenario where the optimal pricing strategy made by the alliance as a price maker will call for medical institutions to respond with their service quality strategy. It is a positive correlation between the treatment equilibrium price and disease treatment PED, i.e., the higher the PED in the treatment of the disease, the higher the price of the treatment. This is the opposite of Ramsey pricing. Medical services can be categorized as basic medical services and special medical services. The PED of basic medical services is low, and it is largely for non-profit-making purposes. In this regard, the pricing for this type of medication will be low. While special medical services have more significant commodity attributes and their high PED value leads the pricing to be higher than that of basic medical services.

To analyze the relationship between the treatment pricing and the coefficient of patients' service quality preference, it is necessary to set the elasticity of treatment demand *b* = 0.3, the parameter o‘f the demand function *a* = 0.3, and the coefficient of treatment quality cost γ = 10.

**Figure 2:**
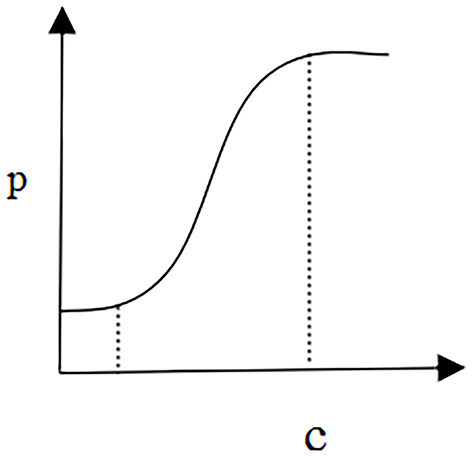
Price and preference.

Figure 2 shows a scenario that reflects the relationship between the equilibrium price of diseases and the coefficient of patients' quality preferences. In this scenario, medical institutions act as price makers. Based on the objective of maximizing profit, their priority is to optimize disease quality control decisions. Furthermore, the alliance of government and patients as the price taker will make the optimal price decision according to the goal of maximizing social welfare. In this model, when the coefficient of patients' quality preferences can be controlled in a certain range, the relationship between disease equilibrium price and patients' quality preference can be positive, i.e., the higher the coefficient of patients' quality preference, the more disease equilibrium price increases, and vice versa.

**Figure 3:**
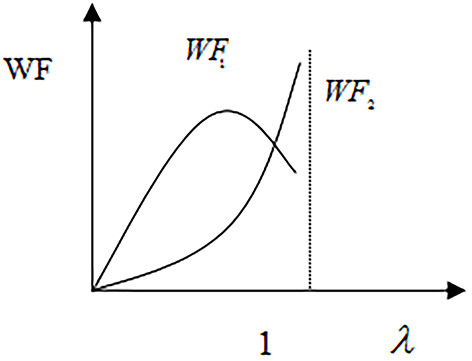
Welfare and co-payment ratio.

The curve *WF*_1_ in Figure 3 shows that the total social welfare increases first and then decreases with the increase of social medical insurance co-payment ratio, where the patients' absolute risk aversion coefficient is $R\geq \frac{\sigma^{2}b+\frac{1}{2}a\delta }{\delta^{2}a}$. The curve *WF*_2_ presents another scenario in which the patients' absolute risk aversion coefficient is $R<\frac{\sigma^{2}b+\frac{1}{2}a\delta }{\delta^{2}a}$and the total social welfare increases with the increase of social insurance co-payment ratio.

The above scenarios reflect the patient's risk aversion attitude toward health threats. When patients have a higher absolute risk aversion coefficient, they will experience more medical moral hazard, and when the co-payment rate of social insurance increases to a certain extent, it will lead to a loss on total social welfare. Opposite to this, when patients' absolute risk aversion coefficient reduces, their medical moral hazard decreases. This can lead to a continuous increase in the total social welfare while the social insurance co-payment ratio increases.

## Conclusion

In the process of DRGs operation in many countries, especially in developing countries, such as India and China, unreasonable pricing and government compensation of medical services under the DRGs operation environment will lead to two major problems. On the one hand, underpricing will lead to medical institutions shirking the responsibility of critically ill patients and decomposing the DRGs category; on the other hand, overpricing will lead to a sharp increase in or even out of control medical expenses. To balance the interests of the government, patients, and medical institutions, the improved bargaining game model based on the Stackelberg game is introduced in order to balance this system and improve the total utility level of society.

The game pricing model developed in this study considers the relationship among the three roles including the government, medical institutions, and patients. The interests and behaviors of these roles have been studied through the models which present a suggestion on price negotiation and setting to the government and medical institutions, and the conducted result can guide medical institutions with a better price-setting strategy. The model also classifies price-setting according to the price elasticity of demand for medical services. To be more realistic, the study has considered price-setting under the impact of patients' preferences for medical service quality. As a result, we find that the absolute risk aversion coefficient of patients affects the relationship between the co-payment rate of medical expenses and total social welfare. In addition, with more investment in public health education, the government can control the moral risk of over-treatment of patients during the operation of DRGs by influencing the absolute risk aversion coefficient of patients and limiting the absolute risk aversion coefficient of patients in a certain range. The future of this study is to generalize these models to DRGs pricing systems with the effects of competition of medical institutions, including considering differences in the compensation of DRGs systems between infirmaries and hospitals, in order to promote the goal of hierarchical diagnosis and treatment.

## Data Availability Statement

The original contributions presented in the study are included in the article/supplementary materials, further inquiries can be directed to the corresponding author/s.

## Author Contributions

JD conceiving the algorithm, designing the model, and writing—original draft. ZL revising and editing. FJ writing and editing. All authors contributed to the article and approved the submitted version.

## Funding

This work was partially supported by the Innovation Strategy Research Project of Fujian Province under grant 2020R0090.

## Conflict of Interest

The authors declare that the research was conducted in the absence of any commercial or financial relationships that could be construed as a potential conflict of interest.

## Publisher's Note

All claims expressed in this article are solely those of the authors and do not necessarily represent those of their affiliated organizations, or those of the publisher, the editors and the reviewers. Any product that may be evaluated in this article, or claim that may be made by its manufacturer, is not guaranteed or endorsed by the publisher.
